# Supplementary figures and images for: ABCH2 transporter mediates deltamethrin uptake and toxicity in the malaria vector Anopheles coluzzii
Source: PLoS Pathog. 2023 Aug 16;19(8):e1011226. doi: 10.1371/journal.ppat.1011226 (PMC10461823; doi:10.1371/journal.ppat.1011226)

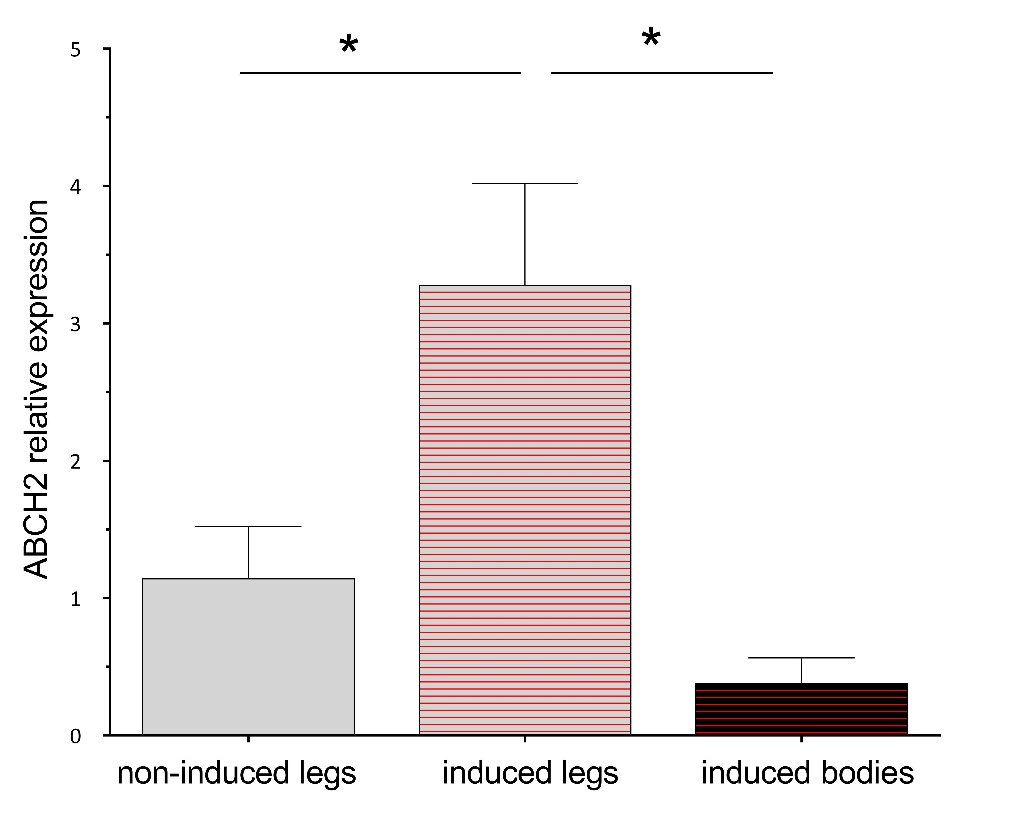

Supplement: S1 Fig — Mean + SEM of three biological replicates per condition (n = 45 per condition). ABCH2 expression is 2.8-fold higher in induced legs, compared to non-induced (P value = 0.0487, *) and 8.6-folds higher in induced legs compared to induced bodies (P value = 0.0192, *). Bodies represent the whole female mosquitoes, lacking the legs. (TIF) [file ppat.1011226.s001.tif]

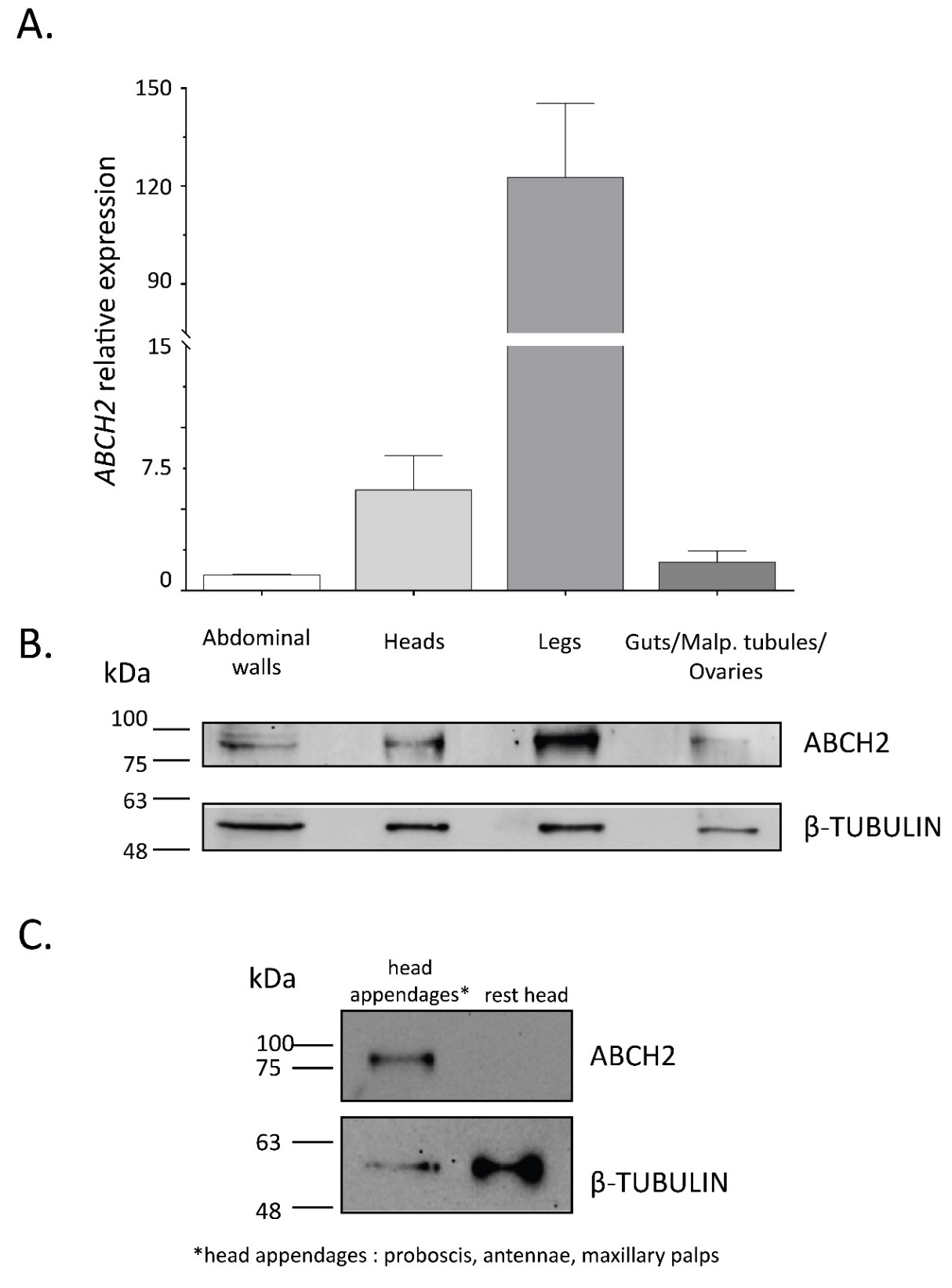

Supplement: S2 Fig — A. Relative expression levels of ABCH2 in different dissected tissues normalized against abdominal walls of 3–5 Day old female VK7 mosquitoes. Bar graphs represent mean values; error bars standard mean error; n = 3 biological replicates and B. Western Blot using specific antibody against ABCH2, depicting ABCH2 in different dissected tissues of 3–5 day old female VK7. Each band corresponds to the tissue of the bar above. C. Western blot analysis indicating ABCH2 presence in head is exclusively attributed to sensory appendages. β-tubulin was used as loading control. (TIF) [file ppat.1011226.s002.tif]

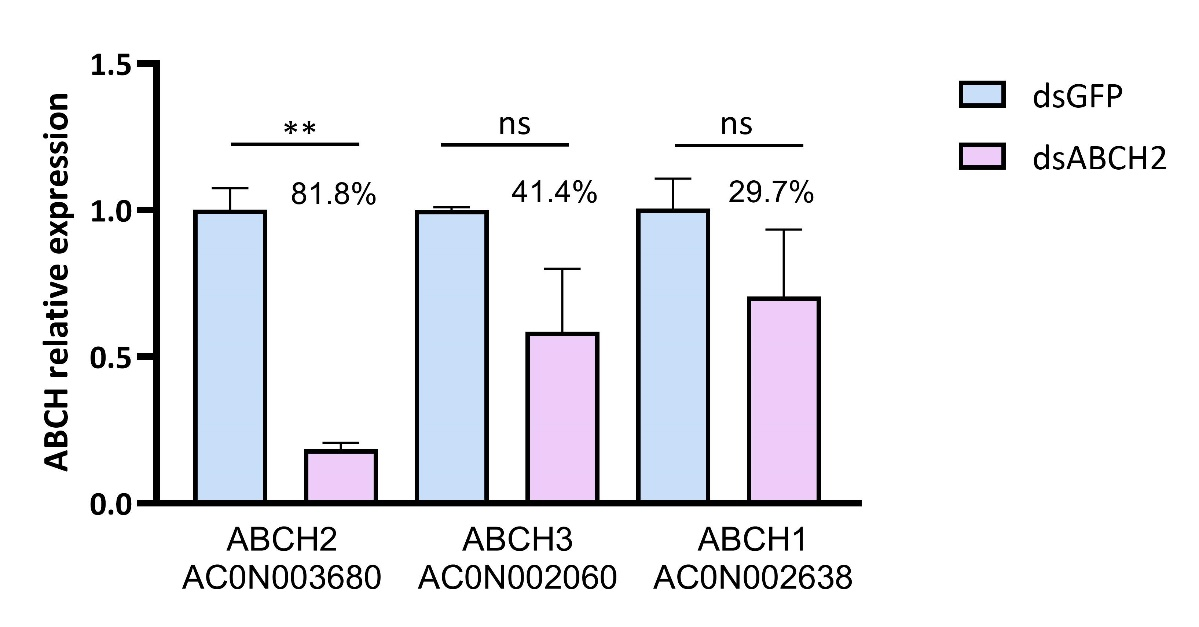

Supplement: S3 Fig — For each gene, expression is normalized against dsGFP. ABCH2 relative expression in dsABCH2-injected mosquitoes is reduced by 81.9% (P-value = 0.0085, **), ABCH3 is reduced by 41.4% (P-value = 0.134, ns) and ABCH1 expression is reduced by 29.7% (P-value = 0.354, ns). (TIF) [file ppat.1011226.s003.tif]

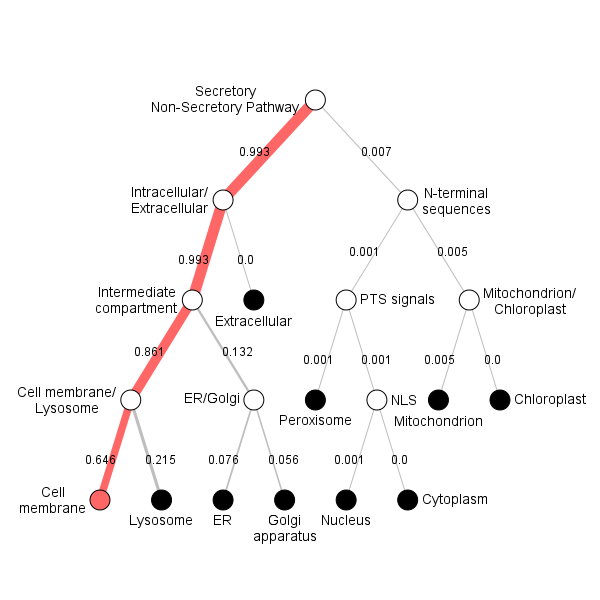

Supplement: S4 Fig — A. Numbers of dsABCH2 and dsGFP mosquitoes exposed in deltamethrin per biological replicate, B. Graphical depiction of total dsABCH2 and dsGFP alive-KD (left panel) and alive-dead (right panel) after 1 hour and 24 hours post deltamethrin exposure respectively, C. Statistical analysis using two-sided exact Fisher’s test: P-value and effect size (odds ratios and reciprocal odds ratio) values are shown for KD and mortality after deltamethrin exposure. (TIF) [file ppat.1011226.s004.tif]

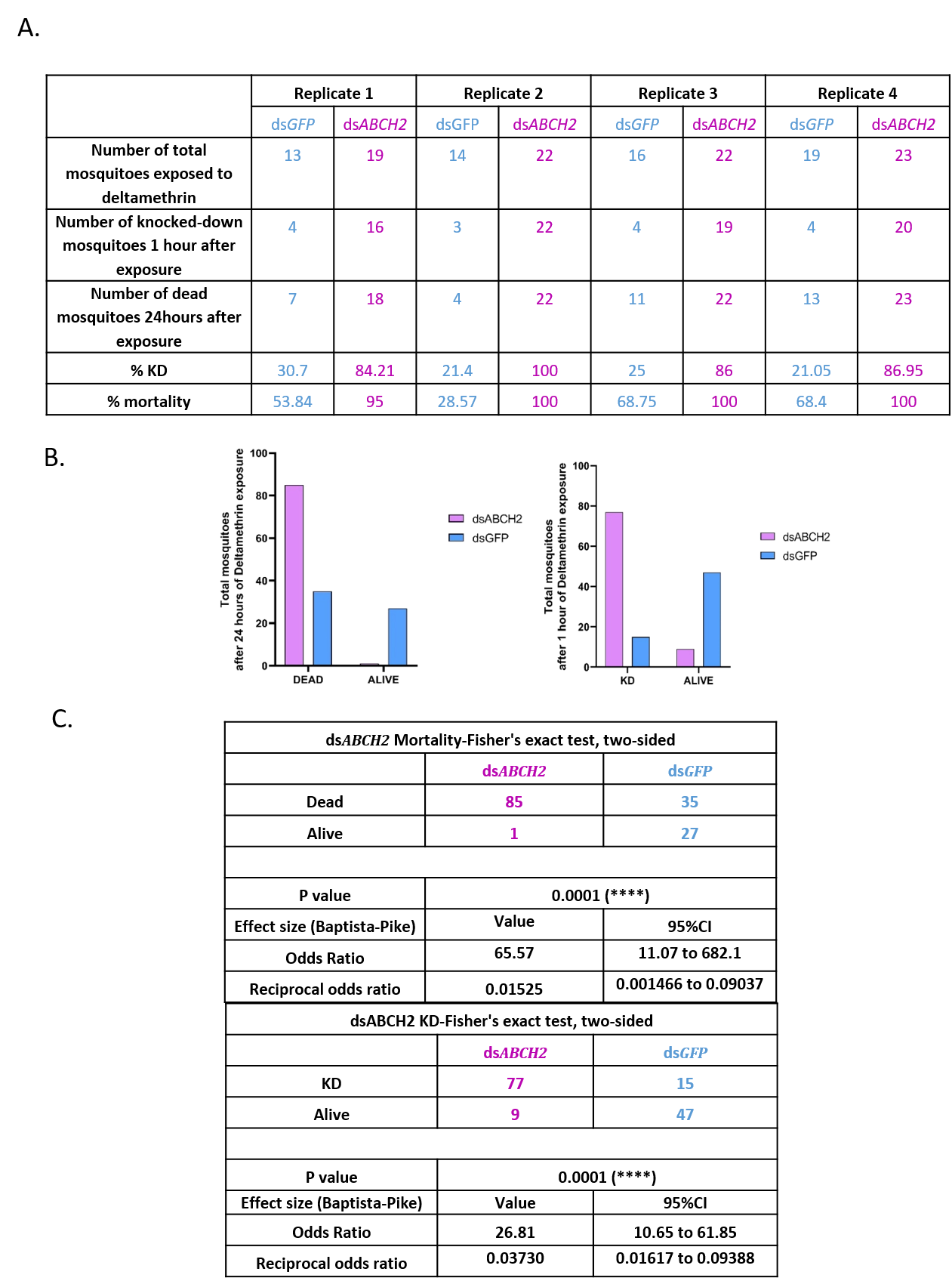

Supplement: S5 Fig — (TIF) [file ppat.1011226.s005.tif]

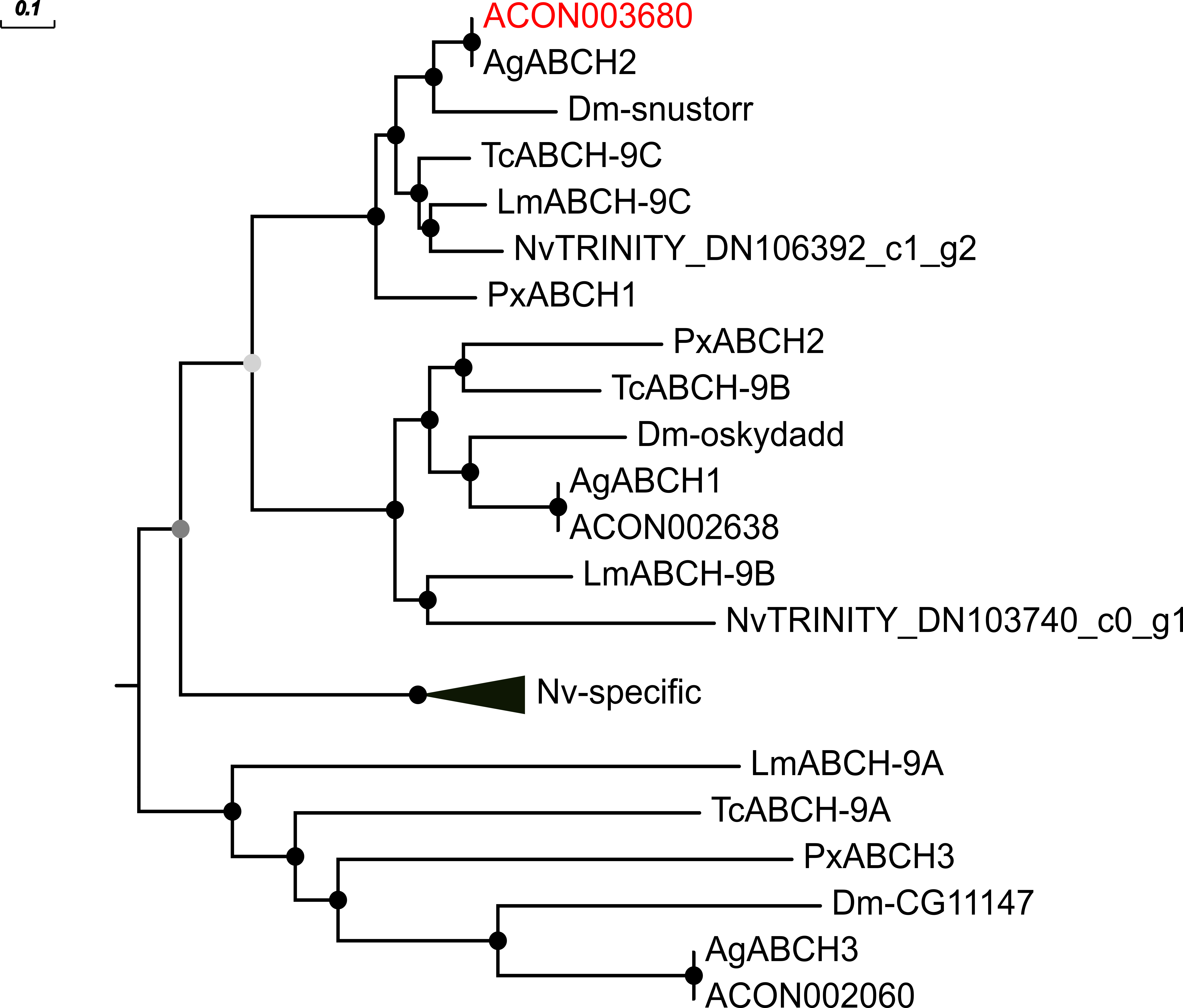

Supplement: S6 Fig — Phylogenetic analysis of ABCH transporters from Anopheles coluzzii (ACON), Anopheles gambiae (Ag), Drosophila melanogaster (Dm), Plutella xylostella (Px), Nezara viridula (Nv), Tribolium castaneum (Tc) and Locusta migratoria (Lm). Tree was created under the LG+R5 substitution model with 5,000 bootstraps and was rooted using the Danio rerio ABCH gene as an outgroup. Nodes with bootstrap support < 50% and between 50% and 75% are indicated with light grey and grey circles respectively. Nodes with bootstrap support greater than 75% are indicated with black circles. (TIF) [file ppat.1011226.s006.tif]

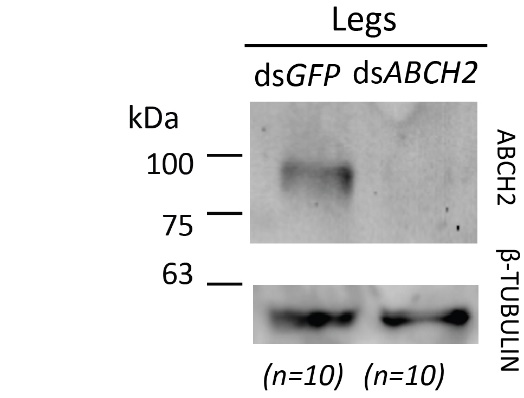

Supplement: S7 Fig — Legs from 10 dsGFP and 10 dsABCH2 were used to validate the silencing. (TIF) [file ppat.1011226.s007.tif]

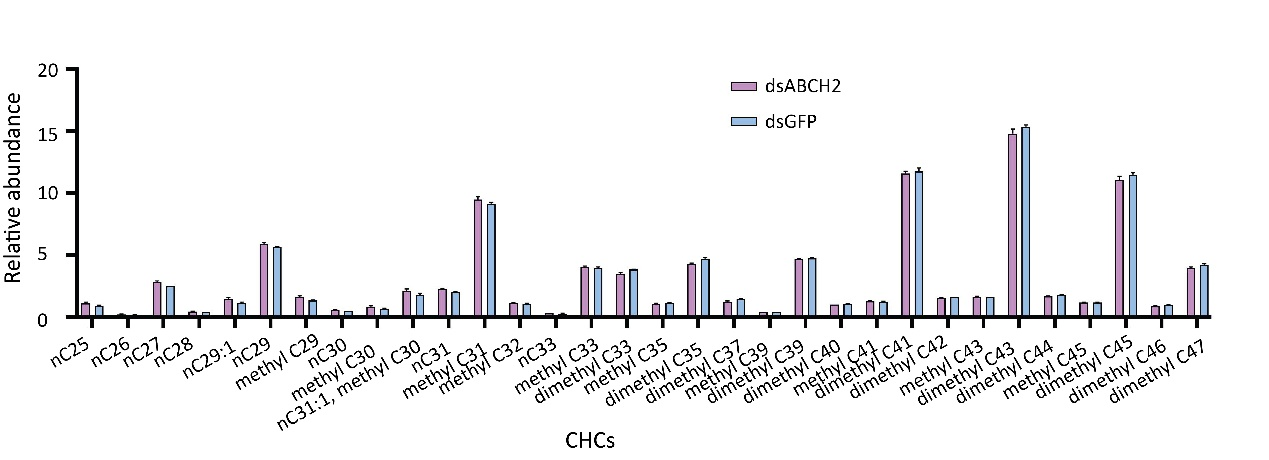

Supplement: S8 Fig — Relative abundances in % area are depicted for each one of the identified CHCs. Mean of 3 biological replicates +SEM. (TIF) [file ppat.1011226.s008.tif]

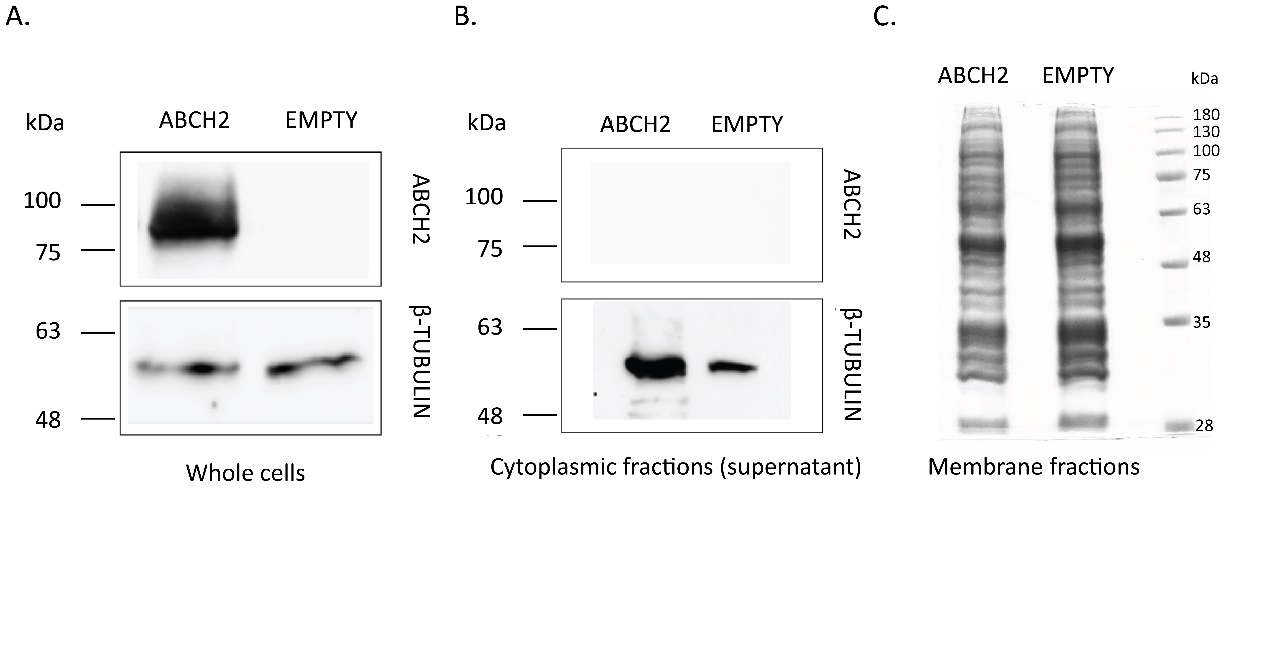

Supplement: S9 Fig — A. Western blot analysis using ABCH2 antibody in whole cell extracts of ABCH2- and Empty Bacmid-infected Sf9 cells, B. Western blot analysis using ABCH2 antibody in cytoplasmic fractions (supernatant). In all cases B-TUBULIN was used as loading control. C. Coomassie blue staining in ABCH2 and Empty membrane fractions. (TIF) [file ppat.1011226.s009.tif]

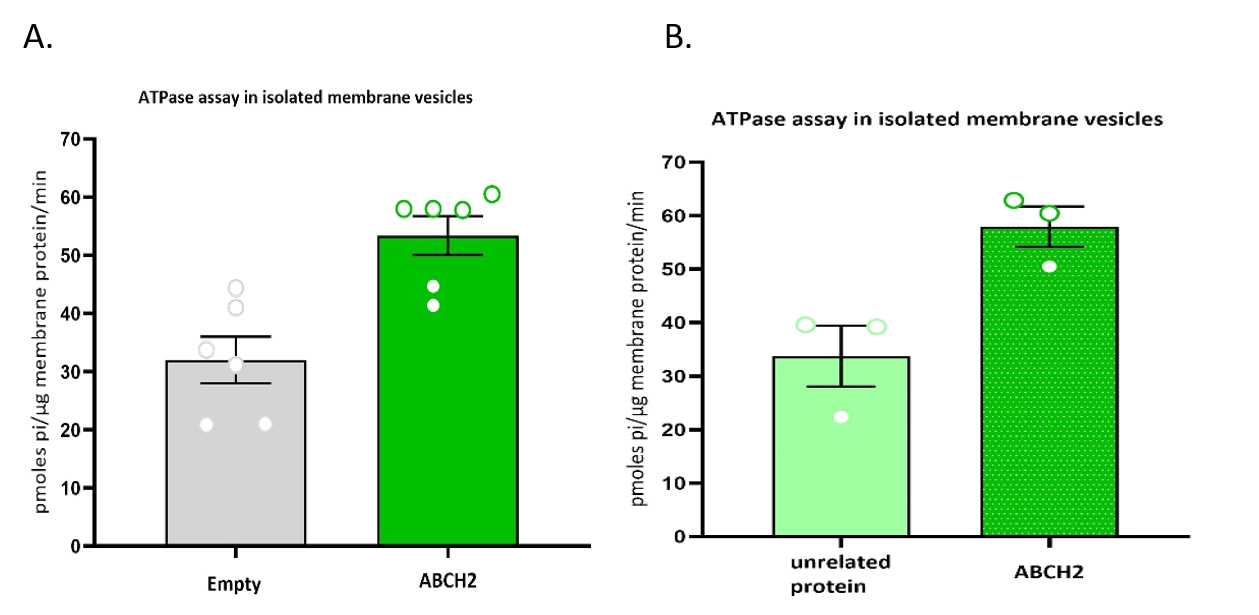

Supplement: S10 Fig — ATPase activity of membrane preparations based on the malachite green colorimetric assay. A. Mean + SEM of six biological replicates in ABCH2 and empty-bacmid expressing membranes. Mean + SEM; Mean(EMPTY) = 32.04+ 4 and Mean(ABCH2) = 52.4+ 3.32 for n = 6 biological replicates; P-value = 0.0021 (**). B. 3 biological replicates in unrelated protein-expressing membranes and ABCH2 membranes. The average of 2–3 technical replicates for each biological is presented in bars. Mean + SEM; Mean(UNRELATED PROTEIN) = 33.74+ 5.67 and Mean(ABCH2) = 57.94+ 3.68; P-value = 0.0238 (*). Empty-bacmid values were used to subtract the Sf9 cell background (Fig 4D). (TIF) [file ppat.1011226.s010.tif]

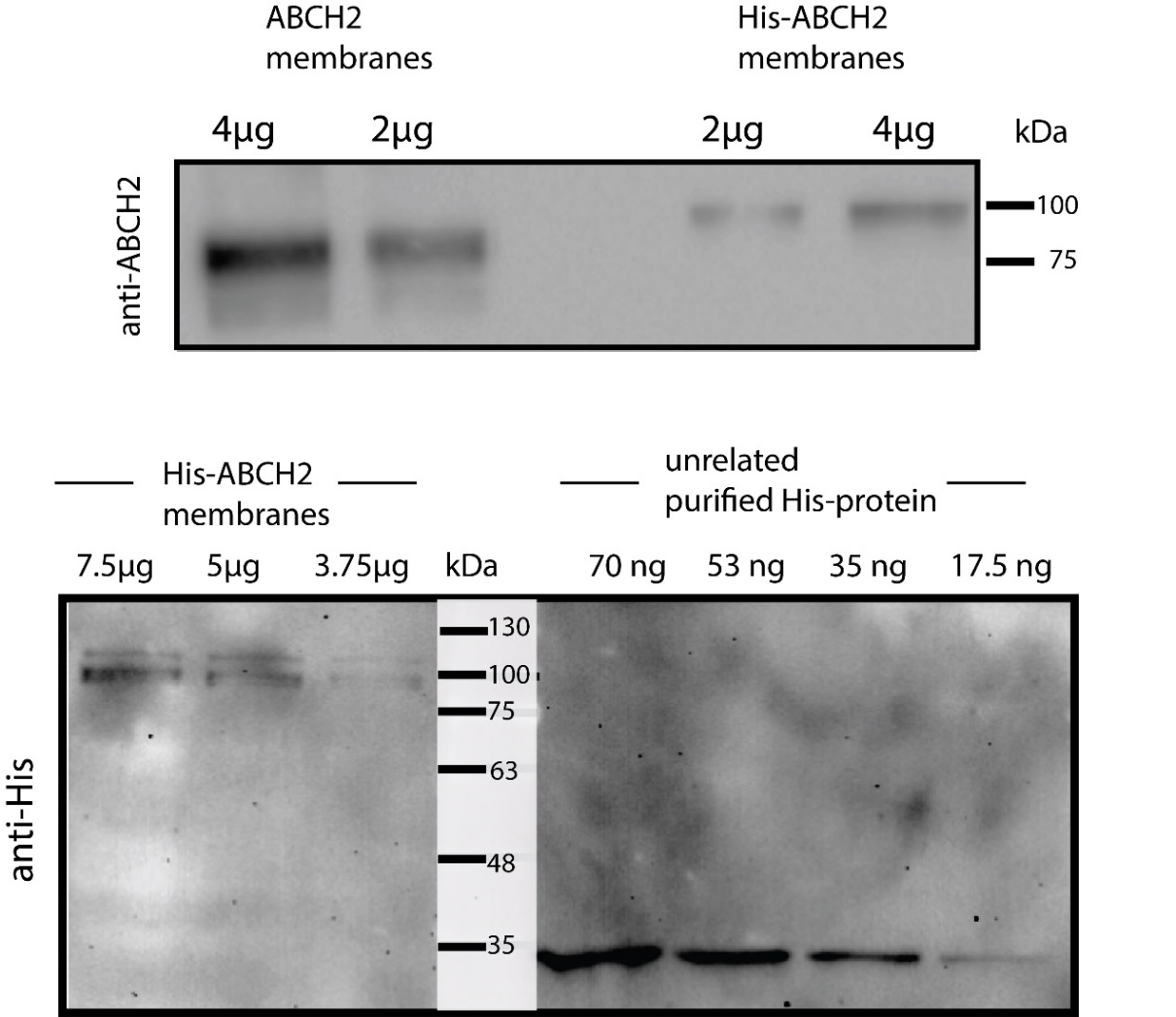

Supplement: S11 Fig — Sf9 infections using ABCH2 and His-ABCH2 bacmids were carried out simultaneously. After isolation of membrane fractions and total protein content estimation using Bradford, the indicated calculated amounts were analyzed by SDS-PAGE and blotted using an ABCH2 specific antibody. At the same time the His-ABCH2 was analyzed simultaneously together with a purified His-tagged control protein to allow estimation of the His-ABCH2 amount using the anti-His antibody. According to densitometry analysis using ImageJ and subsequent calculation (Supplementary file 3), we estimate ABCH2 to be about 11 ng per μgr of total membrane protein, which corresponds to 0.12 pmoles of ABCH2 per μgr of total protein. (TIF) [file ppat.1011226.s011.tif]
