# Supplementary material for: ABCH2 transporter mediates deltamethrin uptake and toxicity in the malaria vector Anopheles coluzzii
Source: PLoS Pathog. 2023 Aug 16;19(8):e1011226. doi: 10.1371/journal.ppat.1011226 (PMC10461823; doi:10.1371/journal.ppat.1011226)
Supplement: S1 File — The standard cut-off distances for all interaction types (hydrophobic, hydrogen-bonds, ionic, aromatic, cation-Pi) were used. The interactions were also manually inspected using Pymol (Molecular Graphics System, version 1.6 Schrödinger, LLC). (PDF) [file ppat.1011226.s017.pdf]

Protein-Protein Hydrophobic Interactions

Hydrophobic Interactions within 5 Angstroms

Position	Residue	Chain	Position	Residue	Chain
172	VAL	A	172	VAL	B
174	VAL	A	203	TYR	B
176	PRO	A	203	TYR	B
176	PRO	A	257	PRO	B
203	TYR	A	174	VAL	B
203	TYR	A	176	PRO	B
257	PRO	A	176	PRO	B
258	ALA	A	177	LEU	B
367	PHE	A	659	LEU	B
371	LEU	A	659	LEU	B
378	LEU	A	666	PRO	B
378	LEU	A	669	LEU	B
378	LEU	A	687	VAL	B
379	PHE	A	665	TYR	B
379	PHE	A	669	LEU	B
381	LEU	A	681	PRO	B
381	LEU	A	684	LEU	B
382	ALA	A	675	TRP	B
382	ALA	A	684	LEU	B
383	ILE	A	675	TRP	B
546	PHE	A	546	PHE	B
547	VAL	A	675	TRP	B
551	VAL	A	675	TRP	B
557	PHE	A	557	PHE	B
557	PHE	A	665	TYR	B
557	PHE	A	668	LEU	B
558	PHE	A	665	TYR	B
561	VAL	A	661	LEU	B
568	LEU	A	657	ILE	B
657	ILE	A	568	LEU	B
657	ILE	A	657	ILE	B
659	LEU	A	367	PHE	B
659	LEU	A	371	LEU	B
661	LEU	A	561	VAL	B
661	LEU	A	661	LEU	B
665	TYR	A	379	PHE	B
665	TYR	A	557	PHE	B
665	TYR	A	558	PHE	B
666	PRO	A	378	LEU	B
668	LEU	A	557	PHE	B
669	LEU	A	378	LEU	B
669	LEU	A	379	PHE	B
669	LEU	A	382	ALA	B
675	TRP	A	382	ALA	B
675	TRP	A	383	ILE	B
675	TRP	A	547	VAL	B
675	TRP	A	551	VAL	B
684	LEU	A	381	LEU	B

TMD-TMD
NBD in yellow

NO PROTEIN-PROTEIN DISULPHIDE BRIDGES FOUND

Protein-Protein Main Chain-Main Chain Hydrogen Bonds

DONOR	ACCEPTOR	PARAMETERS

Dd-a = Distance Between Donor and Acceptor
Dh-a = Distance Between Hydrogen and Acceptor
A(d-H-N) = Angle Between Donor-H-N
A(a-O=C) = Angle Between Acceptor-O=C
MO = Multiple Occupancy
Note that angles that are undefined are written as 999.99

Protein-Protein Main Chain-Side Chain Hydrogen Bonds

DONOR				ACCEPTOR				PARAMETERS				
POS	CHAIN	RES	ATOM	POS	CHAIN	RES	ATOM	MO	Dd-a	Dh-a	A(d-H-N)	A(a-O=C)
145	A	SER	OG	47	B	SER	O	-	3.46	9.99	999.99	150.27
175	A	ASP	N	202	B	HIS	NE2	-	3.11	3.32	69.24	999.99
175	A	ASP	N	203	B	TYR	OH	-	0.71	1.14	38.35	999.99
202	A	HIS	NE2	173	B	GLY	O	-	3.31	3.43	74.98	113.87
203	A	TYR	OH	174	B	VAL	O	-	2.45	9.99	999.99	30.43
203	A	TYR	OH	175	B	ASP	O	-	2.40	9.99	999.99	72.56
364	A	VAL	N	655	B	ASN	OD1	-	3.39	3.62	68.85	136.24
665	A	TYR	OH	554	B	THR	O	-	3.33	9.99	999.99	92.42
145	B	SER	OG	47	A	SER	O	-	3.22	9.99	999.99	143.96
175	B	ASP	N	202	A	HIS	NE2	-	3.42	3.59	71.96	999.99
175	B	ASP	N	203	A	TYR	OH	-	0.40	0.84	22.87	999.99
203	B	TYR	OH	174	A	VAL	O	-	2.55	9.99	999.99	38.79
203	B	TYR	OH	175	A	ASP	O	-	1.99	9.99	999.99	70.17
375	B	GLN	NE2	662	A	GLY	O	1	3.40	3.55	73.04	159.09
375	B	GLN	NE2	662	A	GLY	O	2	3.40	3.57	72.01	159.09
665	B	TYR	OH	554	A	THR	O	-	3.18	9.99	999.99	99.05

NBD-NBD
TMD in yellow

Dd-a = Distance Between Donor and Acceptor
Dh-a = Distance Between Hydrogen and Acceptor
A(d-H-N) = Angle Between Donor-H-N
A(a-O=C) = Angle Between Acceptor-O=C
MO = Multiple Occupancy
Note that angles that are undefined are written as 999.99

Protein-Protein Side Chain-Side Chain Hydrogen Bonds

[View the original hbond output]

model.pdb

DONOR				ACCEPTOR				PARAMETERS				
POS	CHAIN	RES	ATOM	POS	CHAIN	RES	ATOM	MO	Dd-a	Dh-a	A(d-H-N)	A(a-O=C)
139	A	ARG	NH1	25	B	ASN	ND2	1	2.52	3.35	31.45	999.99
139	A	ARG	NH1	25	B	ASN	ND2	2	2.52	1.74	129.84	999.99
139	A	ARG	NH2	25	B	ASN	ND2	1	2.75	3.68	23.89	999.99
139	A	ARG	NH2	25	B	ASN	ND2	2	2.75	2.10	120.15	999.99
175	A	ASP	OD2	261	B	ASN	ND2	1	3.27	2.20	172.91	999.99
175	A	ASP	OD2	261	B	ASN	ND2	2	3.27	3.75	55.36	999.99
203	A	TYR	OH	175	B	ASP	OD1	-	2.84	9.99	999.99	999.99
205	A	GLU	OE2	254	B	ASN	ND2	1	3.23	3.46	68.64	999.99
205	A	GLU	OE2	254	B	ASN	ND2	2	3.23	3.54	64.70	999.99
205	A	GLU	OE2	254	B	ASN	OD1	1	2.93	2.53	100.63	999.99
205	A	GLU	OE2	254	B	ASN	OD1	2	2.93	3.12	69.61	999.99
254	A	ASN	ND2	205	B	GLU	OE1	1	3.40	3.25	89.56	999.99
254	A	ASN	ND2	205	B	GLU	OE1	2	3.40	3.68	66.60	999.99
254	A	ASN	ND2	205	B	GLU	OE2	1	2.91	2.32	114.30	999.99
254	A	ASN	ND2	205	B	GLU	OE2	2	2.91	3.70	34.58	999.99
254	A	ASN	OD1	205	B	GLU	OE2	1	2.87	2.27	113.24	999.99
254	A	ASN	OD1	205	B	GLU	OE2	2	2.87	3.62	38.15	999.99
261	A	ASN	ND2	175	B	ASP	OD2	1	3.03	2.02	161.38	999.99
261	A	ASN	ND2	175	B	ASP	OD2	2	3.03	3.58	49.88	999.99
653	A	GLU	OE1	653	B	GLU	OE2	1	2.97	2.29	119.41	999.99
653	A	GLU	OE1	653	B	GLU	OE2	2	2.97	3.87	26.40	999.99
653	A	GLU	OE2	653	B	GLU	OE1	1	2.83	2.57	92.49	999.99
653	A	GLU	OE2	653	B	GLU	OE1	2	2.83	2.93	74.20	999.99
653	A	GLU	OE2	653	B	GLU	OE2	1	2.59	1.76	130.56	999.99
653	A	GLU	OE2	653	B	GLU	OE2	2	2.59	3.32	38.97	999.99
139	B	ARG	NH1	25	A	ASN	ND2	1	2.95	3.84	27.16	999.99
139	B	ARG	NH1	25	A	ASN	ND2	2	2.95	2.19	129.40	999.99
139	B	ARG	NH2	25	A	ASN	ND2	1	2.78	3.63	31.13	999.99
139	B	ARG	NH2	25	A	ASN	ND2	2	2.78	1.99	133.16	999.99
175	B	ASP	OD2	261	A	ASN	ND2	1	3.03	1.97	169.31	999.99
175	B	ASP	OD2	261	A	ASN	ND2	2	3.03	3.52	53.98	999.99
203	B	TYR	OH	175	A	ASP	OD1	-	2.72	9.99	999.99	999.99
205	B	GLU	OE1	254	A	ASN	ND2	1	3.40	3.58	72.05	999.99
205	B	GLU	OE1	254	A	ASN	ND2	2	3.40	4.03	47.39	999.99
205	B	GLU	OE2	254	A	ASN	ND2	1	2.91	3.11	69.81	999.99
205	B	GLU	OE2	254	A	ASN	ND2	2	2.91	3.21	64.35	999.99
205	B	GLU	OE2	254	A	ASN	OD1	1	2.87	2.39	105.63	999.99
205	B	GLU	OE2	254	A	ASN	OD1	2	2.87	3.02	71.80	999.99
254	B	ASN	ND2	205	A	GLU	OE2	1	3.23	2.66	114.47	999.99
254	B	ASN	ND2	205	A	GLU	OE2	2	3.23	4.05	32.16	999.99
254	B	ASN	OD1	205	A	GLU	OE2	1	2.93	2.27	117.49	999.99
254	B	ASN	OD1	205	A	GLU	OE2	2	2.93	3.59	43.98	999.99
261	B	ASN	ND2	175	A	ASP	OD2	1	3.27	2.25	163.42	999.99
261	B	ASN	ND2	175	A	ASP	OD2	2	3.27	3.90	45.26	999.99
653	B	GLU	OE1	653	A	GLU	OE2	1	2.83	2.21	114.22	999.99
653	B	GLU	OE1	653	A	GLU	OE2	2	2.83	3.69	29.66	999.99
653	B	GLU	OE2	653	A	GLU	OE1	1	2.97	2.80	88.17	999.99
653	B	GLU	OE2	653	A	GLU	OE1	2	2.97	3.09	73.53	999.99
653	B	GLU	OE2	653	A	GLU	OE2	1	2.59	1.90	118.84	999.99
653	B	GLU	OE2	653	A	GLU	OE2	2	2.59	3.34	37.57	999.99

NBD-NBD
TMD in yellow

Dd-a = Distance Between Donor and Acceptor
Dh-a = Distance Between Hydrogen and Acceptor
A(d-H-N) = Angle Between Donor-H-N
A(a-O=C) = Angle Between Acceptor-O=C
MO = Multiple Occupancy
Note that angles that are undefined are written as 999.99

Protein-Protein Ionic Interactions

Ionic Interactions within 6 Angstroms

Position	Residue	Chain	Position	Residue	Chain
175	ASP	A	202	HIS	B
202	HIS	A	175	ASP	B
572	ARG	A	653	GLU	B
653	GLU	A	572	ARG	B

NBD
TMD yellow

Protein-Protein Aromatic-Aromatic Interactions

Aromatic-Aromatic Interactions within 4.5 and 7 Angstroms

Residue	Position	Chain	Residue	Position	Chain	D(centroid-centroid)	Dihedral Angle
546	PHE	A	546	PHE	B	4.55	57.71
557	PHE	A	557	PHE	B	5.41	96.41
557	PHE	A	665	TYR	B	5.68	43.21
558	PHE	A	665	TYR	B	4.61	41.70
665	TYR	A	557	PHE	B	5.74	42.27
665	TYR	A	558	PHE	B	4.95	42.48

TMD-TMD

NO PROTEIN-PROTEIN AROMATIC-SULPHUR INTERACTIONS FOUND

Protein-Protein Cation-Pi Interactions

Cation-Pi Interactions within 6 Angstroms

Position	Residue	Chain	Position	Residue	Chain	D(cation-Pi)	Angle
203	TYR	A	179	ARG	B	4.76	23.73
203	TYR	B	179	ARG	A	4.47	21.36

NBD-NBD

Protein-Protein Hydrophobic Interactions

Hydrophobic Interactions within 5 Angstroms

TMD-TMD

Position	Residue	Chain	Position	Residue	Chain
409	VAL	A	552	VAL	B
410	LEU	A	555	PHE	B
413	LEU	A	552	VAL	B
413	LEU	A	555	PHE	B
413	LEU	A	556	VAL	B
421	ILE	A	559	VAL	B
421	ILE	A	562	ILE	B
421	ILE	A	563	PRO	B
424	LEU	A	563	PRO	B
424	LEU	A	566	LEU	B
424	LEU	A	567	PHE	B
424	LEU	A	584	MET	B
425	ILE	A	566	LEU	B
427	LEU	A	578	PRO	B
427	LEU	A	580	TYR	B
427	LEU	A	581	LEU	B
428	LEU	A	566	LEU	B
428	LEU	A	567	PHE	B
428	LEU	A	572	VAL	B
428	LEU	A	577	ILE	B
428	LEU	A	578	PRO	B
428	LEU	A	581	LEU	B
429	TYR	A	566	LEU	B
429	TYR	A	572	VAL	B
430	LEU	A	578	PRO	B
447	PHE	A	571	PHE	B
448	PHE	A	562	ILE	B
448	PHE	A	565	LEU	B
448	PHE	A	566	LEU	B
448	PHE	A	571	PHE	B
451	LEU	A	451	LEU	B
451	LEU	A	562	ILE	B
451	LEU	A	565	LEU	B
451	LEU	A	571	PHE	B
452	PHE	A	559	VAL	B
452	PHE	A	562	ILE	B
455	PHE	A	455	PHE	B
455	PHE	A	558	PRO	B
455	PHE	A	562	ILE	B
456	ALA	A	555	PHE	B
456	ALA	A	559	VAL	B
459	MET	A	459	MET	B
459	MET	A	555	PHE	B
459	MET	A	558	PRO	B
460	PRO	A	555	PHE	B
463	LEU	A	463	LEU	B
463	LEU	A	550	LEU	B
467	LEU	A	550	LEU	B
550	LEU	A	463	LEU	B
550	LEU	A	467	LEU	B
550	LEU	A	550	LEU	B
552	VAL	A	409	VAL	B
552	VAL	A	413	LEU	B
555	PHE	A	410	LEU	B
555	PHE	A	413	LEU	B
555	PHE	A	456	ALA	B
555	PHE	A	459	MET	B
555	PHE	A	460	PRO	B
556	VAL	A	413	LEU	B
558	PRO	A	455	PHE	B
558	PRO	A	459	MET	B
559	VAL	A	421	ILE	B
559	VAL	A	452	PHE	B
559	VAL	A	456	ALA	B
562	ILE	A	421	ILE	B
562	ILE	A	425	ILE	B
562	ILE	A	448	PHE	B
562	ILE	A	451	LEU	B
562	ILE	A	452	PHE	B
562	ILE	A	455	PHE	B
563	PRO	A	421	ILE	B
563	PRO	A	424	LEU	B
565	LEU	A	448	PHE	B
565	LEU	A	451	LEU	B
566	LEU	A	424	LEU	B
566	LEU	A	425	ILE	B
566	LEU	A	428	LEU	B
566	LEU	A	429	TYR	B
566	LEU	A	448	PHE	B
567	PHE	A	424	LEU	B
567	PHE	A	428	LEU	B
570	PHE	A	570	PHE	B
570	PHE	A	571	PHE	B
571	PHE	A	447	PHE	B
571	PHE	A	448	PHE	B
571	PHE	A	570	PHE	B
572	VAL	A	428	LEU	B
572	VAL	A	429	TYR	B
577	ILE	A	428	LEU	B
578	PRO	A	428	LEU	B
578	PRO	A	430	LEU	B
580	TYR	A	427	LEU	B
581	LEU	A	427	LEU	B
581	LEU	A	428	LEU	B
584	MET	A	424	LEU	B

NO PROTEIN-PROTEIN DISULPHIDE BRIDGES FOUND

Protein-Protein Main Chain-Main Chain Hydrogen Bonds

DONOR **ACCEPTOR** **PARAMETERS**

Dd-a = Distance Between Donor and Acceptor
Dh-a = Distance Between Hydrogen and Acceptor
A(d-H-N) = Angle Between Donor-H-N
A(a-O=C) = Angle Between Acceptor-O=C
MO = Multiple Occupancy
Note that angles that are undefined are written as 999.99

Protein-Protein Main Chain-Side Chain Hydrogen Bonds

NBD-NBD

DONOR				ACCEPTOR				PARAMETERS				
**POS**	**CHAIN**	**RES**	**ATOM**	**POS**	**CHAIN**	**RES**	**ATOM**	**MO**	**Dd-a**	**Dh-a**	**A(d-H-N)**	**A(a-O=C)**
248	A	ASP	N	274	B	HIS	NE2	-	3.30	3.57	66.57	999.99
274	A	HIS	NE2	246	B	GLY	O	-	2.83	3.01	69.89	140.10
275	A	GLN	NE2	247	B	LEU	O	1	3.21	3.32	74.75	152.02
275	A	GLN	NE2	247	B	LEU	O	2	3.21	2.83	102.17	152.02
218	B	SER	OG	120	A	SER	O	-	3.33	9.99	999.99	149.71
221	B	GLN	NE2	120	A	SER	O	1	3.45	3.71	67.33	124.63
221	B	GLN	NE2	120	A	SER	O	2	3.45	2.63	135.31	124.63
274	B	HIS	NE2	246	A	GLY	O	-	3.05	3.27	68.10	133.76
275	B	GLN	NE2	247	A	LEU	O	1	3.49	3.58	76.95	147.62
275	B	GLN	NE2	247	A	LEU	O	2	3.49	3.04	107.73	147.62

Dd-a = Distance Between Donor and Acceptor
Dh-a = Distance Between Hydrogen and Acceptor
A(d-H-N) = Angle Between Donor-H-N
A(a-O=C) = Angle Between Acceptor-O=C
MO = Multiple Occupancy
Note that angles that are undefined are written as 999.99

Protein-Protein Side Chain-Side Chain Hydrogen Bonds

NBD-NBD
TMD in yellow

DONOR				ACCEPTOR				PARAMETERS				
**POS**	**CHAIN**	**RES**	**ATOM**	**POS**	**CHAIN**	**RES**	**ATOM**	**MO**	**Dd-a**	**Dh-a**	**A(d-H-N)**	**A(a-O=C)**
249	A	SER	OG	323	B	ASP	OD2	-	3.18	9.99	999.99	999.99
252	A	CYS	SG	275	B	GLN	OE1	-	3.71	9.99	999.99	999.99
275	A	GLN	OE1	252	B	CYS	SG	1	3.69	3.32	101.27	999.99
275	A	GLN	OE1	252	B	CYS	SG	2	3.69	3.79	76.08	999.99
279	A	LYS	NZ	323	B	ASP	OD1	-	3.43	9.99	999.99	999.99
279	A	LYS	NZ	323	B	ASP	OD2	-	3.17	9.99	999.99	999.99
407	A	ASP	OD2	551	B	GLN	OE1	1	2.54	2.17	97.33	999.99
407	A	ASP	OD2	551	B	GLN	OE1	2	2.54	2.29	90.91	999.99
551	A	GLN	OE1	407	B	ASP	OD2	1	2.69	1.92	125.46	999.99
551	A	GLN	OE1	407	B	ASP	OD2	2	2.69	3.02	61.62	999.99
249	B	SER	OG	323	A	ASP	OD2	-	2.77	9.99	999.99	999.99
252	B	CYS	SG	275	A	GLN	OE1	-	3.69	9.99	999.99	999.99
275	B	GLN	OE1	252	A	CYS	SG	1	3.71	3.34	101.72	999.99
275	B	GLN	OE1	252	A	CYS	SG	2	3.71	3.92	70.70	999.99
279	B	LYS	NZ	323	A	ASP	OD2	-	3.37	9.99	999.99	999.99
407	B	ASP	OD2	551	A	GLN	OE1	1	2.69	2.24	102.97	999.99
407	B	ASP	OD2	551	A	GLN	OE1	2	2.69	2.41	93.34	999.99
551	B	GLN	OE1	407	A	ASP	OD2	1	2.54	1.84	119.10	999.99
551	B	GLN	OE1	407	A	ASP	OD2	2	2.54	2.73	68.24	999.99

Dd-a = Distance Between Donor and Acceptor
Dh-a = Distance Between Hydrogen and Acceptor
A(d-H-N) = Angle Between Donor-H-N
A(a-O=C) = Angle Between Acceptor-O=C
MO = Multiple Occupancy
Note that angles that are undefined are written as 999.99

Protein-Protein Ionic Interactions

Ionic Interactions within 6 Angstroms

NBD-NBD

Position	Residue	Chain	Position	Residue	Chain
165	ASP	A	222	ARG	B
222	ARG	A	165	ASP	B
248	ASP	A	274	HIS	B
274	HIS	A	248	ASP	B
279	LYS	A	323	ASP	B
279	LYS	A	327	GLU	B
323	ASP	A	279	LYS	B
327	GLU	A	279	LYS	B

Protein-Protein Aromatic-Aromatic Interactions

Aromatic-Aromatic Interactions within 4.5 and 7 Angstroms

Residue	Position	Chain	Residue	Position	Chain	D(centroid-centroid)	Dihedral Angle
318	TYR	A	318	TYR	B	6.76	109.67
447	PHE	A	571	PHE	B	6.36	15.65
448	PHE	A	571	PHE	B	4.68	63.53
455	PHE	A	455	PHE	B	4.66	125.87
570	PHE	A	570	PHE	B	6.18	101.56
570	PHE	A	571	PHE	B	6.12	177.61
571	PHE	A	447	PHE	B	6.50	15.37
571	PHE	A	448	PHE	B	5.01	64.10
571	PHE	A	570	PHE	B	5.94	178.17

TMD-TMD
NBD in yellow

NO PROTEIN-PROTEIN AROMATIC-SULPHUR INTERACTIONS FOUND

NO PROTEIN-PROTEIN CATION-PI INTERACTIONS FOUND
