# Supplementary material for: ABCH2 transporter mediates deltamethrin uptake and toxicity in the malaria vector Anopheles coluzzii
Source: PLoS Pathog. 2023 Aug 16;19(8):e1011226. doi: 10.1371/journal.ppat.1011226 (PMC10461823; doi:10.1371/journal.ppat.1011226)
Supplement: Attachment — Submitted filename: Letter_Responce to Reviewers.pdf [file ppat.1011226.s018.pdf]

11 July 2023

PLOS Pathogens

Ref: *PPATHOGENS-D-23-00324 - [EMID:602af3fca5cf176c]*

Dear Editor,

Thank you very much for the excellent reviews, which have very much helped us to improve our manuscript. Please find below a detailed response to all comments and suggestions made by the Referees.

We trust our manuscript can now be accepted for publication in PloS Pathogens

Sincerely,

John Vontas

Part I- Summary

Reviewer #1

This MS describes the functional study of an ABC-transporter (ABCH2) from the malaria vector Anopheles coluzzi. In particular, the potency of this ABC-transporter to mediate the toxicity of deltamethrin (a pyrethroid insecticide widely used on bednets) is investigated through a series of descriptive and functional experiments.

First, using RT-qPCR and western blotting, the authors showed that ABCH2 is strongly expressed in mosquito legs and head appendices as compared to other tissues, in line with its potency to interact with the insecticide following an epidermal/tarsal contact. Then, in vivo RNAi silencing (intrathoracic injection of ABCH2 dsRNA into adult females) was used to show that ABCH2 expression is associated with an increased deltamethrin survival and a decreased C14-deltamethrin penetration. Immunofluorescence was then used to show that ABCH2 protein is mainly localized in the sub-cuticular epithelia of legs and antennae. Comparing total cuticular hydrocarbon (CHCs) content and CHCs composition between dsABCH2- and dsGFP-injected mosquitoes did not support the hypothesis of ABCH2 being involved in CHC deposition. In silico modelling and docking simulations supported ABCH2 acting as a homo-dimeric active transmembrane deltamethrin transporter. In vitro experiments using recombinant ABCH2 showed an increased ATPase activity in presence of deltamethrin supporting its ability to actively transport the insecticide. Altogether, these experiments support the role of this ABC-transporter in mediating deltamethrin toxicity in the malaria vector An. coluzzii.

Overall, this MS presents a huge amount of work and provides interesting functional data about the role of ABC transporters in mediating insecticide toxicity in malaria vectors. The MS is well written, figures are well designed and supplementary materials are overall informative.

Reply: We thank the reviewer for the positive comments.

However, I found the whole story slightly over-sold with some experimental data possibly over-interpreted (see specific comments below). This feeling is also supported by a very straight forward discussion which, in my view, does not always mention the technical biases potentially affecting some of the experimental data presented and their interpretation. The discussion would also benefit from an additional section replacing the findings into the broader context of the response/adaptation of mosquitoes to insecticides. For instance, discussing the relative importance of this mechanism versus

other known insecticide resistance mechanisms and its adaptive value (as opposed to a non-adaptive effect in mediating insecticide toxicity) would be of interest for the community.

Reply: We thank the reviewer for this comment. We added a new paragraph in the discussion sections: Lines: **424-436 (lines refer to the revised version with tracked changes)**

.Reviewer #2:

While malaria vector control still relies heavily on the use of insecticides deployed as long-lasting insecticidal nets and indoor residual spraying, insecticide resistance increasingly threatens the sustainability of current mosquito control programmes. Understanding the mechanisms conferring insecticide resistance in the malaria vector may help to find new targets in the development of alternative active ingredients. One of the mechanisms of insecticide resistance is resistance due to reduction in insecticide uptake through the insect's cuticle, which is the topic of the present article. Specifically, the study investigated the role of the ATP-Binding-Casette (ABC) transporter ABCH2 in conferring resistance to the pyrethroid insecticide deltamethrin in the sub-Saharan malaria vector, Anopheles coluzzii. The methods used are a combination of many complementary approaches, including RNAi-mediated silencing of ABCH2 in a pyrethroid-resistant A. coluzzii mosquitoes from a laboratory colony, standard insecticide susceptibility assays to characterise the phenotype of silenced and control groups, measurements of 14C-labelled deltamethrin between silenced and control groups, immunolocalisation of ABCH2 in legs and head appendages with immunostaining and confocal microscopy, measurement of cuticular hydrocarbons contents between the groups, determination of gene expression levels using RT-qPCR, use of in silico protein models as well as ATPase activity measurements in heterologously expressed ABCH2. The overall emerging picture from the results is that deltamethrin is a substrate of ABCH2 that then actively export deltamethrin through hydrolysis of ATP.

A strength of the study is its holistic approach to understand the mechanism of ABCH2 in conferring resistance to deltamethrin in A. coluzzii. Where I found the study is less convincing is to what degree ABCH2 plays a role in shaping the resistance phenotype. While the main conclusion of the study is that ABCH2 is a 'key' regulator of deltamethrin toxicity, I feel the data do not show to what extent ABCH2 indeed plays a role in deltamethrin detoxification since effect sizes are not provided and some of the experiments lack sufficient replication (see comments below). Therefore, it may not be concluded that

ABCH2 is a ‘key regulator of deltamethrin toxicity. Nevertheless, the study highlights an intriguing aspect of insecticide detoxification in mosquitoes and opens the door for further studies and, as such, it is novel and merits sharing with the wider research community.

Reply: We thank the reviewer for positive evaluation of our study and the very helpful and constructive comments.

we changed the title, as recommended, and rephrased the last abstract phrase sentence (**line 35**). We also added discussion parts that expands the study perspectives, including clarifications for possible limitations and future work **406-423**

(lines refer to the revised version with tracked changes).

Part II – Major Issues: Key Experiments Required for Acceptance
Please use this section to detail the key new experiments or modifications of existing experiments that should be absolutely required to validate study conclusions.

Reviewer #1:

Considering that several experiments rely on the differential mortality observed between dsABCH2- and dsGFP-injected mosquitoes, I suggest strengthening the presentation of these data. For instance, it is mentioned that there are 3 ABCH transporters in An. coluzzi (with high sequence homology) while the specificity of dsABCH2 against other ABCH transporters is not presented. Considering the RT-qPCR data presented, I believe that adding this does not represent a major challenge as the same cDNA samples as those used for ABCH2 expression data can be used. From the method section, I understand that total RNA was extracted from legs so it might not be possible to assess the effect of dsABCH2-injection on all three ABCH transcripts in various tissues, though at least presenting such data for legs will be informative. This is a major point because providing such data will eliminate any doubt regarding the specificity of the phenotype observed upon dsABCH2 injection and strengthen subsequent experiments and general conclusions. In addition, although the mortality difference between dsGFP- and dsABCH2-injected mosquitoes appears significant, the underlying raw data should be made available to the reader (as supplementary information) in order to support the statistical approach presented. Indeed, Fig 1 does not show replicate data points (only means and total number of mosquitoes), and Fig1 legend does not precise if SEM or SD are shown. The way experiment was replicated should also be better described in the method section (lines 478-481): It is mentioned ‘14-22 individuals x 4 replicates per dsRNA’, but not sure what is considered as a replicate (same injected-

mosquito batch, or totally independent injection experiments with a different mosquito batch?) and how many mosquitoes per tube were used per replicate. Again, providing raw data as supplementary information (number mosquitoes KD, dead, alive for each test tube and each replicate) will strengthen the study. In the same line but of lesser importance, the experiment looking at CHCs deposition also involves comparing dsABCH2- and dsGFP-injected mosquitoes. As this experiment does not show any significant difference between the two conditions (thus allowing to reject the hypothesis of ABCH2 being involved in CHC deposition), one would expect these CHC data to be backed up with RT-qPCR data showing a significant knock down of ABCH2 expression on the same injected mosquito batch. If not technically possible, this should be mentioned in the results and/or the discussion as this may affect data interpretation

Reply: We thank the reviewer for this comment. Considering recommendations of both the reviewers we have added five supplementary figures to strengthen the presentation of the data and we have also fixed the representation of the figures in the main text:

For **Figures 1A, 1C, 1D** and **Figure 3** individual values are shown in the revised manuscript. Additionally, in response to reviewer 2 we present figures **1A**, **1C** and **1D** with 95% confidence intervals. Moreover, for mortality and knock-down (**Figures 1C and D**) we have added the information of the biological samples in lines: **536-543** (main text) and Figure **S4A** including the raw data of this experiment. In response to reviewer 2 recommendation, the statistical analysis was performed with an exact Fisher's test and the statistics are presented in detail in Figure **S4B, C**. For Figure **1E** we have added an extra biological replicate and we include the raw data of C-14-deltamethrin counts (external, internal and total) in supplementary **table S4A**, along with the statistical analysis details in **table S4B**. The figure legend of Figure **1** was changed accordingly. Additionally, regarding the CHC data of Figure **3**, the silencing efficiency of the batch of injected mosquitoes was estimated by western blot analysis (Supplementary Figure **S8**) and the information is now included in lines **596-597.**

Regarding the specificity of the dsRNA construct against the two other ABCH transporters, we also tested that in freshly-prepared leg samples (Figure **S2**), and we added lines **149-151** and **538-541.**

Reviewer #2:

A key experiment, which I find is very important to substantiate ABCH2's role in deltamethrin detoxification, is the measurement of the change in deltamethrin content between ABCH2 silenced and

the GFP control mosquitoes using 14C-deltamethrin. It is unfortunate that data from only two replicates per group are available which makes it impossible to estimate the effect size of silencing ABCH2 (i.e. the difference reported is statistically not significant). As this is a key experiment to make a strong case for the function and role of ABCH2, the manuscript would substantially improve by repeating this assay with more replicates and estimating the effect size.

Reply: Thank you. We have performed a third biological replicate (Figure 1E). The raw data from C-14 counts of all replicates, along with the statistical analysis are presented in Supplementary **Table S4**.

Similar experimental design issues are present in other parts of the study. Although the difference between CHC of legs from dsABCH2 and dsGFP may be small, making a claim that CHS is not affected by ABCH2 silencing based on a sample size of 3 is very bold. In this case, I would argue that a lack of evidence is not an evidence of lack as the experiment is hugely underpowered. Therefore, either more replicates are needed to make such a statement - together with a power calculation to indicate what difference may be detected - or the authors ought to be more cautious in the interpretation of these results.

We changed the title of this section to: "Deltamethrin toxicity **could not** be attributed to CHC differences in the legs" (**line 216**).

This is a biological material limitation issue, rather than an experimental design issue.

Briefly there are 3 replicates in this experiment. A technical challenge was that the analysis was carried out in dried legs and we needed at least 1μg of tissue per replicate to proceed to FID-MS. To achieve this quantity, we needed legs of about 30-35 injected mosquitoes in each biological replicate and hence the data are represented by about 100 mosquitoes in each case. Additionally, the relative abundance of each hydrocarbon species identified by the analysis, presented in the supplementary **Figure S8** also supports that there is not a significant difference in any identified CHC species and that these three replicates group quite well (small deviations in all CHC species). Deviations between the three replicates in both conditions (total average and each individual CHC species) are also small and hence we considered it is safe to draw this conclusion. Lastly, a silencing validation of the injected mosquito batch is also provided in the revised supporting information file 1 **(Figure S8 - lines 226-227and 596-597)**. Of course, we cannot exclude the possibility that this small reduction in the average of total CHC amount, even though not significant, could impact on deltamethrin penetration, and in the revised manuscript we discussed this and have added appropriate caveats to the "evidence of lack" conclusion.

Limitations and more cautious interpretations are also discussed in the submitted revised manuscript in lines: **416-423**

Finally, I would like to see a critical discussion of alternative hypothesis and weaknesses of the study in the Discussion section. For instance, is it feasible that ABCH2 plays an important role (other than detoxification of deltamethrin) that would lead to higher mortality when silenced? Where do the authors feel the study has weaknesses in terms of the methods applied?

Reply: Thank you. In To address this point we added a new discussion paragraph: lines **406-423**

Part III – Minor Issues: Editorial and Data Presentation Modifications
Please use this section for editorial suggestions as well as relatively minor modifications of existing data that would enhance clarity.

Reviewer #1: **- Title: The title seems a bit overselling as the MS does not show any evidence that the mechanism studied differentiates resistant from susceptible populations.**

Consider changing to something more realistic such as 'ABCH2 transporter mediates deltamethrin uptake and toxicity in the malaria vector An. coluzzi'.

Reply: The title was changed as recommended

- line 54-55: ref (8) repeated twice

Reply: Thank you. We corrected that in the MS.

- line 73: TBD -> TMD?

Reply: Yes, we meant to write TMD. Thank you for the correction

line 82: Maybe I am mistaken, but I think that ref (6) and (10) do not support the over-transcription of ABCH2 in insecticide resistant populations nor its induction after insecticide exposure. The following sentence was taken from ref (10): 'The down-regulated transcripts include a number of cuticular proteins and the ABC transporter ABCH2, a half transporter whose role in insects is poorly characterised [18]'. This looks as overselling the rational of the study.

Reply: Yes, we agree that ABCH2 is not over-transcribed in multiple datasets. The sentence in line 82 supports that ABC transporters of H family have been found differentially expressed in several insect datasets. Reference 6 and 10 include mosquito studies, while the rest reference ABCH transporters of other insects. ABCH2 was found induced after 1 hour of deltamethrin exposure in the leg dataset of ref 6 that is why it is cited there. Regarding ref 10, ABCH2 was found to be down-regulated 12 hours after deltamethrin exposure.

To avoid any misunderstanding we rephrase these two paragraphs: lines **98-115**

- line 87: I did not find mention of ABCH expression in ref (4).

Reply: ABCH2 (AGAP003680) is identified in the leg proteome. This data are represented in supplementary file 2 in excel format (rspb20191091supp2.xlsx). Apart from AGAP003680, no other ABC transporters were identified in this dataset.

In addition, in ref (6) the An. coluzzi ortholog of An. gambiae ABCH2 (see the phylogenetic analysis in supplementary material) was not overexpressed in legs (as opposed to ABCH3), neither induced by deltamethrin (as opposed to ABCG11, ABCE2, ABCC2 and BCG2) or associated with constitutive deltamethrin resistance (no ABC transporter mentioned). Again, this looks as overselling the rational of the study. In this regard, a screening of the recent literature in An. gambiae/coluzzi for a synthesis of ABCH gene expression data in regards of insecticide resistance and xenobiotic exposure may be of interest as this may consolidate the rational of the study but also feed the discussion about the role of mosquito ABCH transporters in their response to insecticides.

We plot here the TPM expression values of the three ABCH transporter genes in the relevant conditions (Fig. 1, raw data from Kefi & Charamis et al., 2021).

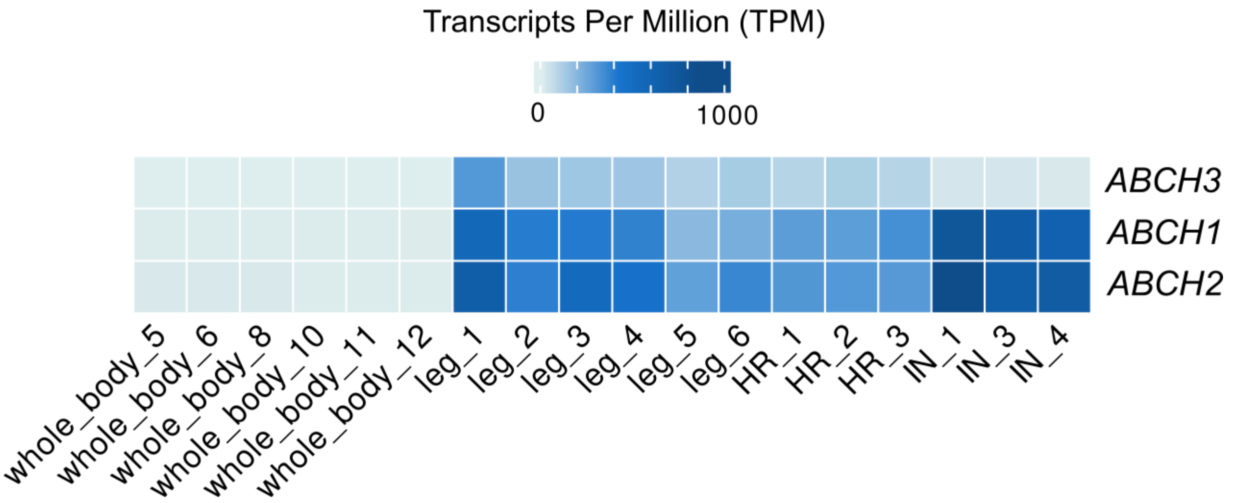

Figure 1. Normalized values of *ABCH1*, *ABCH2*, *ABCH3* absolute gene expression in susceptible whole-body and legs, as well as in resistant (HR) and 1hr deltamethrin-exposed resistant legs (IN) from Kefi & Charamis et al. (2021).

From this heatmap you can see that:

All three ABCH genes are enriched in susceptible legs vs the whole body. *ABCH1* and *ABCH2* were not reported as DE in Kefi & Charamis et al. 2021,BMC Genocmics, because of statistical filtering of the genes with extreme DE values. From the Methods section of the paper: "*For discovery of transcripts showing leg*

specific expression, filterByExpr was used to remove genes with low counts as described, DREAM was then used to fit a linear mixed model taking into account both the population and the leg compared to whole organism whilst reducing false positive rate. *ABCH2* and *ABCH1* are more highly expressed than *ABCH3* both in susceptible and resistant legs. *ABCH2* and *ABCH1* are ~1.7 times more highly expressed in resistant legs after deltamethrin exposure, while *ABCH3* is not (Table 1).

Table 1. Results of DE analysis VK7-IN vs VK7-HR (induced vs resistant legs) from Kefi & Charamis et al. (2021).

**Gene**	**log2FC VK7 (IN vs HR)**	**P-value**	**FDR**
ABCH2 (ACON003680)	1.744	5.412e-45	3.97e-43
ABCH1 (ACONP002638)	1.646	1.94e-42	1.28e-40
ABCH3 (ACON002060)	-1.756	1.31e-17	2.14e-16

We proceeded with *ABCH2* and not *ABCH1*, because it was the only identified ABCH transporter in the leg proteome of resistant *An. gambiae* (Balabanidou et al., 2019). Search for "AGAP003680" in attached supplementary file 2 (rspb20191091supp2.xlsx).

Finally, we also confirmed the induction in legs with RT-qPCR (Fig. 2):

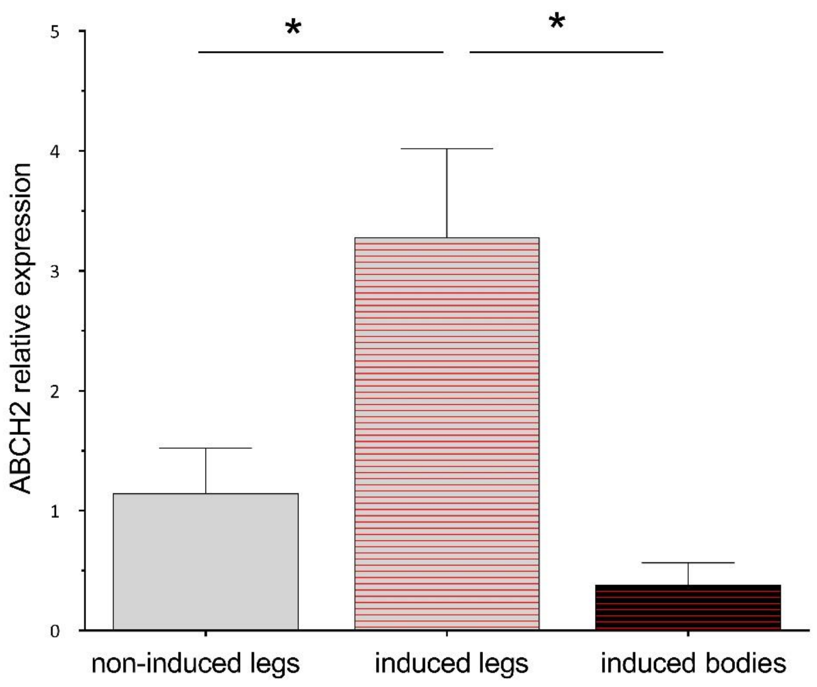

Figure 2. ABCH2 relative expression in non-induced legs, 1hour-deltamethrin induced legs and bodies. Mean + SEM of three biological replicates per condition (n=45 per condition). ABCH2 expression is 2.8 fold higher in induced legs, compared to non-induced (P value= 0.0487, *) and 8.6 folds higher in induced legs compared to induced bodies (P value =0.0192, *). Bodies represent the whole female mosquitoes, lacking the legs.

This figure was added as supplementary **Figure 1S**, and lines (**127-128**) refer to this in the text.

- lines 95-96: same comment as above (line 87).

Thank you, we have deleted and rephrased this part.

- Fig4D. Why not mentioning which protein was used as control?

We used a cytochrome P450 protein (*An. gambiae* CYP4G16), which is anchored to the membranes with a single N-terminal transmembrane domain and has no transport or ATPase activity.

We added this sentence in the materials and method part, line **628-631**

line 392: Explain the rational of using the VK7-LR colony (instead of the VK7 colony). You may also want to describe the remaining resistance mechanisms identified in this colony based on previous work (kdr mutation frequency, other, …).

The VK7 strain (Namountougou et al., 2012, Plos One and Williams et al.,2019, Parasites and Vectors), was initially colonized, but could not be maintained under insecticide selection probably due to the high fitness cost. At the time the study was conducted, the VK7-LR (maintained without insecticide exposure for several generation) was the only available *An. coluzzii strain* colonized in the laboratory and the LC50 (0.016%) was evaluated as described in Kefi & Charamis et al.,2021.

Reviewer #2: Generally, the manuscript has many typos. I recommend careful revision of the text by the native Anglophone co-authors.

Reply: Thank you. We rephrased/corrected many typos.

Spell out the scientific names on first mentioning. For example, line 50 ('Anopheles coluzzii'), line 59 ('Bemisia tabaci' and 'Plutella xylostella').

Reply: Thank you. We corrected this.

Lines 116 – 120 and Figure 1C and D. Using Student's t-test to compare mortality rates is unsuitable. Use a binomial test, instead. It would be useful to know the odds ratios incl. 95% confidence intervals to have an idea of the effect size

Reply: Thank you for this comment. We performed two-sided Fisher's exact test for both mortality and knock-down and added the following information in the legend of Figure 1 and in the **Figure 4S B and C.**

Figure 1A: This figure seems a bit odd to me. I would have expected either only one bar chart with the fold changes in ABCH2 compared to dsGFP or two bars with the actual expression levels. The number of mosquitoes in the brackets is not readable. I would also suggest showing the 95% confidence limits rather than SEM as it is more intuitive.

Reply: Thank you for the comment. We analyzed the dsABCH2 samples against dsGFP. In that way the depiction shows that dsGFP expression is about "1" and dsABCH2 is around 0.25 relatively to that. In that case the Y axis should be named "relative expression" and not "relative fold change". We corrected that, included the 95% CI, added the individual values of each replicate, and also corrected the brackets. Since we performed again the silencing estimation experiment in freshly prepared samples we now merged this data with the previous silencing efficiency data, to improve the statistics. In the supplementary **Figure S3** only the values corresponding to the new samples tested are plotted, to allow silencing estimation comparisons between the ABCH2, ABCH1 and ABCH3, tested in the same experiment.

Figure 1C and D: see comments with regards to the statistical analysis above. I am not too familiar with RNAi experiments. Is it usual that we expect such high mortality rates in the controls? Or is the laboratory colony a rather susceptible one (i.e. the insecticide dose chosen too high)? Please provide an explanation in the text / discussion.

Reply: The dose we used here is the LC50 for the VK7-LR strain (Kefi & Charamis et al.,2021, BMC Genomcis). Thus,the mortality in the dsGFP control is as expected, around 50%.

Figure 1E: The supposed difference between dsGFP and dsABCH2 is inflated because the y-axis does not start at 0%. Adjust the y-axis with 0% as the lower limit.

Reply: Regarding Figure 1E, we have added an extra biological replicate. In **Table S4** we provide all the information on the replicates, the number of mosquitoes, the external, internal and total deltamethrin counts, the % penetration and the statistics. Based on these changes, we believe it might be acceptable to represent y-axis from a higher limit than the 0% recommended, to allow a more focused look of the data, which are now plotted with different shapes corresponding to each biological replicate.

Figure 2: Increase the size of the labels on the images. The scale bars, for example, cannot be read. Consider reducing the figure to make it fit on one page (e.g. removing the panel with the bright field only).

Reply: Thank you for the recommendation. We removed the bright-field channel and increased the size of the scale bars and labels.

Line 297 – 298: Delete 'the slower insecticide penetration rate, provided by'.

Reply: Agree, deleted.

Line 309: I would suggest changing 'could suggest' to 'suggests'.

Reply: Agree, corrected, thank you.

Materials and methods: This section is technically a bit heavy, which is just the nature of this kind of work. However, it would help the reader if the sections were more structured into paragraphs and sentences summarising the steps at the beginning of a paragraph.

Reply: Thank you. We added one summarizing sentence in each material and method paragraph.

Line 402, 'Dissected tissues': Remind the reader what tissues were dissected.
Reply: Thank you, we used a parenthesis to include this information accordingly.

Line 418, '20 tissues': I assume you meant to write '20 tissue samples' or '20 samples per tissue type'.

Reply: We apologize for the misunderstanding. We meant to write that we prepared three replicates per tissue, with each replicate containing 20 dissected tissues. The amount of trizol used to extract these 20 tissues was 200 ul.

We rephrased **lines 482-483**

Line 423, '[company]': Is something missing here?

Reply: The company of the reverse transcriptase used in the study is "Minotech Biotechnology". We corrected that, thank you.

Line 444: Maybe better to write '40x magnification lense' instead of 'objective'?
Reply: Thank you, we corrected that.

Line 486, '0.01% 14C-deltamethrin paper'. This needs a bit more explanation here. What method was used to treat the paper? Did it follow the WHO methods, i.e. same type of filter paper? Either provide a reference to the method in the published literature or describe how the papers were prepared.

Reply: Thank you. We prepared the paper using standard WHO bioassay protocols. We added this information and the reference (**lines 568-571**)

Line 506: Explain how the dry weight was 'calculated'.

Reply: Thank you. We used a balance scale of μg (micro scale). Legs of dried mosquitoes were pooled and measured in micro scale. Each replicate was about 1 μg and the number of mosquitoes used for that were also recorded to allow us to normalize per mosquito.

Lines:593-596

Line 605: By 'fork' do you mean 'sub company'?

Reply: By fork (autodock smina) we refer to repository that shares code and visibility settings with the original "upstream" (autodock vina) repository. We need to mention both developer parties for accuracy.
